# The Sero-epidemiology of *Neospora caninum* in Cattle in Northern Tanzania

**DOI:** 10.3389/fvets.2019.00327

**Published:** 2019-09-26

**Authors:** George Semango, Clare M. Hamilton, Katharina Kreppel, Frank Katzer, Tito Kibona, Felix Lankester, Kathryn J. Allan, Kate M. Thomas, John R. Claxton, Elizabeth A. Innes, Emmanuel S. Swai, Joram Buza, Sarah Cleaveland, William A. de Glanville

**Affiliations:** ^1^Nelson Mandela African Institution of Science and Technology, Tengeru, Tanzania; ^2^Moredun Research Institute, Pentlands Science Park, Edinburgh, United Kingdom; ^3^Paul G. Allen School for Global Animal Health, Washington State University, Pullman, WA, United States; ^4^Institute of Biodiversity, Animal Health and Comparative Medicine, University of Glasgow, Glasgow, United Kingdom; ^5^Centre for International Health, Dunedin School of Medicine, University of Otago, Dunedin, New Zealand; ^6^Good Samaritan Foundation, Kilimanjaro Clinical Research Institute, Moshi, Tanzania; ^7^Ministry of Livestock and Fisheries, Dodoma, Tanzania

**Keywords:** Tanzania, *Neospora caninum*, livestock-husbandry, prevalence, risk factors, reproductive loss

## Abstract

*Neospora caninum* is a protozoan intracellular parasite of animals with a global distribution. Dogs act as definitive hosts, with infection in cattle leading to reproductive losses. Neosporosis can be a major source of income loss for livestock keepers, but its impacts in sub-Saharan Africa are mostly unknown. This study aimed to estimate the seroprevalence and identify risk factors for *N. caninum* infection in cattle in northern Tanzania, and to link herd-level exposure to reproductive losses. Serum samples from 3,015 cattle were collected from 380 households in 20 villages between February and December 2016. Questionnaire data were collected from 360 of these households. Household coordinates were used to extract satellite derived environmental data from open-access sources. Sera were tested for the presence of *N. caninum* antibodies using an indirect ELISA. Risk factors for individual-level seropositivity were identified with logistic regression using Bayesian model averaging (BMA). The relationship between herd-level seroprevalence and abortion rates was assessed using negative binomial regression. The seroprevalence of *N. caninum* exposure after adjustment for diagnostic test performance was 21.5% [95% Credibility Interval (CrI) 17.9–25.4]. The most important predictors of seropositivity selected by BMA were age greater than 18 months [Odds ratio (OR) = 2.17, 95% CrI 1.45–3.26], the local cattle population density (OR = 0.69, 95% CrI 0.41–1.00), household use of restricted grazing (OR = 0.72, 95% CrI 0.25–1.16), and an increasing percentage cover of shrub or forest land in the environment surrounding a household (OR = 1.37, 1.00–2.14). There was a positive relationship between herd-level *N. caninum* seroprevalence and the reported within-herd abortion rate (Incidence Rate Ratio = 1.03, 95% CrI 1.00–1.06). Our findings suggest *N. caninum* is likely to be an important cause of abortion in cattle in Tanzania. Management practices, such as restricted grazing, are likely to reduce the risk of infection and suggest contamination of communal grazing areas may be important for transmission. Evidence for a relationship between livestock seropositivity and shrub and forest habitats raises questions about a potential role for wildlife in the epidemiology of *N. caninum* in Tanzania.

## Introduction

Neosporosis, caused by an obligate intracellular protozoan parasite, *Neospora caninum*, is a livestock disease with worldwide distribution. The parasite causes disease in cattle and small ruminants, with cycles involving domestic dogs (*Canis lupus familiaris*), coyotes (*Canis latrans*), and the Australian dingo (*Canis lupus dingo*) as definitive hosts reported (1, 2). Cattle can become infected when they feed on pastures contaminated by wild or domestic canine feces containing sporulated *Neospora* oocysts (3). Transmission can also occur trans-placentally when a cow is infected during pregnancy or following the reactivation of a latent infection in a pregnant animal (4, 5). In cattle, the parasite causes abortions, stillbirths, neonatal deaths, early fetal loss, and embryo reabsorption (3, 6) with reproductive losses usually observed during the second trimester of pregnancy. The parasite can also cause disease early in gestation which may increase the calving interval or present as infertility (7). Congenital infection can also lead to the birth of weak, premature calves, or calves with neurological disease, or they may be born with no obvious clinical signs. Global economic losses due to neosporosis to the beef and dairy industries are estimated at up to one billion US dollars annually (3, 8). *Neospora caninum* is therefore regarded as a major, economically important pathogen of cattle (8). Recent reports suggest that *N. caninum* can also cause disease in small ruminants (9–12), however the potential economic impacts are yet to be assessed.

Despite the economic importance of neosporosis in cattle, there are no treatments or vaccines currently commercially available. Prevention and control therefore relies on reducing exposure of cattle to infectious *N. caninum* oocysts (13), culling out seropositive dams, or restricting breeding to sero-negative dams (3). Reported risk factors for *N. caninum* infection in cattle include the presence of dogs in cattle-keeping households, history of abortion, herd size, hygiene practices (14), handling of abortus (15), introduction of new cattle to the herd (16), grazing practices (17), and production system (3). The positive association with dog ownership has been found to be further increased when dogs have access to cattle placentas and fetuses (18, 19).

The reported seroprevalence of *N. caninum* exposure in cattle ranges between 7.6 and 41% in the Americas (15, 20), 10.7 and 19.6% in Africa (14), 4.1 and 43% in Asia (21, 22), 0.5 and 27.7% in Europe (23), and 10.2% in Oceania (24). These data may not be directly comparable due to differences in serological methods and cut-off values used, but they do provide evidence of the global distribution of the parasite (3). In East Africa, *N. caninum* seropositivity was recently found in 17.9% of farm dogs and 25.6% of cattle in the Nakuru District of Kenya, with exposure in farm dogs associated with free-roaming (25). In Kenya, serological evidence for *N. caninum* infections has also been reported in wild animals, including zebra (*Equus quagga*), eland (*Taurotragus oryx*), buffalo (*Syncerus caffer*), gazelle (*Gazella thomsonii*), impala (*Aepyceros melampus*), and warthog (*Phacochoerus africanus*), as well as spotted hyena (*Crocuta crocuta*) and cheetah (*Acinonyx jubatus*) (26).

Little is currently known about the epidemiology and impacts of *N. caninum* in Tanzania. This study aimed to establish the seroprevalence in cattle across northern Tanzania and to quantify the association between *N. caninum* seropositivity and a range of potential risk factors. To explore disease impacts, we also assessed the relationship between the rate of cattle abortions within a herd and the within-herd seroprevalence of *N. caninum* exposure.

## Methods

### Study Design

Livestock samples and household questionnaire data were collected as part of the “Social, Economic and Environmental Drivers of Zoonotic disease” (SEEDZ) study (grant no. BB/L018926/1). The methods have been described elsewhere (27). Briefly, this was a cross-sectional survey conducted in six districts in Arusha Region (Arusha, Karatu, Longido, Meru, Monduli, and Ngorongoro Districts) and four districts in Manyara Region (Babati Rural, Babati Urban, Mbulu, and Simanjaro Districts), Tanzania, between February and December 2016. The study involved quantitative and qualitative data collection and was designed with a target sample of 400 households in order to address a range of questions relating to zoonotic disease transmission. A multistage sampling design was used, with village as the primary sampling unit. Twenty villages were selected from a spatially referenced list of all villages in the study area (from the Tanzanian National Bureau of Statistics) using generalized random tessellation stratified sampling (28). Livestock sampling was conducted at two to three sites within each village using a central point approach, with livestock owners invited to bring animals to a pre-selected point by notifying them of the event through traditional village-level communication routes (i.e., a network of village elders) at least 24 h before the event. Central point sampling events were run in collaboration with the Tanzanian Ministry of Livestock and Fisheries as part of village-level disease control activities, including the provision of anthelminthics. Up to 10 households were selected at random from all who attended each central point event using a random number generator. Ten cattle were randomly selected per household in order to detect infection with 90% confidence assuming a within-herd prevalence of 25% (29). Cattle <6 months of age were excluded from the sample.

Ten milliliters of blood were collected using jugular venipuncture into plain vacutainers. Samples were allowed to clot before serum extraction on the day of collection. Cattle were aged by dentition. Within 1 week, livestock keepers were visited in their homes and a questionnaire was conducted with the household head. Questions focused on household demographics, economics, livestock management and livestock health. Household co-ordinates were collected using a handheld GPS (Garmin eTrex, Garmin Ltd, Olathe, Kansas, USA). Pre-tested household surveys were conducted in Kiswahili or Maa using Open Data Kit data collection software (https://opendatakit.org/) on tablet computers.

### Ethical Approval

All participants whose animals were sampled and who completed questionnaires provided written informed consent. The protocols, questionnaires and consent procedures were approved by the ethical review committees of the Kilimanjaro Christian Medical Centre (KCMC/832) and National Institute of Medical Research (NIMR/2028) in Tanzania, and in the UK by the ethics review committee of the College of Medical, Veterinary and Life Sciences, University of Glasgow. Approval for the animal elements of the study was provided by the Clinical Research Ethics Committee at the University of Glasgow School of Veterinary Medicine (39a/15). Permission to publish this manuscript was granted by the Director of Veterinary Services, Tanzania.

### Serological Testing

Serum samples were heat treated at 56°C for 2 h prior to export for serological testing. Testing was performed at the Moredun Research Institute, UK, using an in-house ELISA.

#### Preparation of Recombinant *Neospora caninum* SRS2

Forward (5′ tcg gta ccg gtg tcg ggt gcg ccg ttc aag 3′) and reverse (5′ atc ccg ggt cag tac gca aag attg ccg ttgc 3′) primers were designed for the *N. caninum* SRS2 antigen gene. The primers were used to amplify a region of the gene SRS2 that encodes amino acids 20 to 354. The PCR amplicon was cloned directionally into the pQE31 expression vector (QIAGEN, UK) using restriction enzymes *Kpn*I and *Xma*I. After confirming the validity of the expression clone by sequence analysis, the construct was used to express and purify the recombinant His-tagged *N. caninum* SRS2 antigen in the *E. coli* strain M15, containing plasmid pREPp4, following the QIA*expressionist*™ (QIAGEN) instructions.

#### Detection of *Neospora caninum* Antibodies in Cattle Sera

Microwells of 96-well medium binding plates (Greiner Bio-One, UK) were coated at 4°C overnight with recombinant *N. caninum* SRS2 antigen (amino acids 20-354) at a concentration of 0.5 μg/ml in 0.1 M sodium carbonate buffer. Following washing, wells were blocked for 1 h at 37°C with 4% Marvel dried milk powder diluted in phosphate buffered saline containing 0.05% Tween-20 (PBST). Plates were washed and control and test sera were added in duplicate at a dilution of 1:500 in 2% Marvel diluted in PBST and incubated for 2 h at 37°C. Following washing, Horse Radish Peroxidase-conjugated rabbit anti-bovine IgG (Sigma, UK) was added at a dilution of 1:2000 in PBST and incubated for 2 h at 37°C before washing and the addition of substrate (tetramethylbenzidine). Reactions were stopped by the addition of 2M H_2_SO_4_ and the optical density of each plate was measured at 450 nm using a microplate reader. Duplicate samples of positive and negative control sera were included on each plate. The positive control sample was pooled sera from three cows from a farm in Scotland which had each suffered an abortion, were positive for *Neospora* antibodies with a commercial ELISA, and in which histopathology indicated neosporosis. The negative control sample was pooled sera from three cows which had no history of *N. caninum* infection and which were negative with a commercial ELISA. Test thresholds for defining positive and negative results on the basis of ELISA sample to positive (S/P) ratios were determined using a bimodal latent class mixture model implemented within a Bayesian framework, as previously described (30, 31). The resulting S/P ratio cut-off that maximized diagnostic sensitivity and specificity was 18.3, with an estimated sensitivity of 74.3% (95% CrI 67.3–81.3) and specificity of 95.7% (95% CrI 93.6–97.5). Given limited information on the epidemiology of *N. caninum* in cattle in Tanzania, we also derived an S/P ratio threshold of 25 which maximized specificity [99.9% (95% CrI 99.8–1.0)] at the expense of sensitivity [58.7% (95% CrI 50.3–66.7)] (31). This higher threshold ensured a high level of confidence in positive results, particularly given the low to moderate expected seroprevalence in the region (32–34). We used this conservative threshold for inference, and include results derived using the more liberal threshold for reference.

### Statistical Analyses

#### Prevalence Estimation

The “observed” prevalence estimates at both the conservative (24) and liberal (18.3) S/P thresholds were adjusted by diagnostic specificity and sensitivity in order to derive “true” prevalence estimates (35). Adjustment for diagnostic test performance was performed using the *prevalence* package (36) in the R statistical environment version 3.6.0 (37).

#### General Contextual Analysis

Given the hierarchical nature of the study design, in which sampled cattle were clustered by household and village, we first performed a general contextual analysis to examine the relative effects of these grouping-levels in explaining variation in the odds of *N. caninum* seropositivity (38). A null logistic regression model was run with random effects at the household- and village-level but without fixed effects. The median odds ratio (MOR) and intra-cluster correlation coefficient (ICC) were calculated using the estimated variance in household- and village-level intercepts. The MOR provides an estimate of the magnitude of heterogeneity in odds of infection at each level while the ICC provides an estimate of the correlation in infection probability at each level (39). The ICC was estimated using the latent variable approach (40). We also examined whether the residual log odds of infection at the village-level showed evidence of spatial autocorrelation using the Moran's I statistic.

#### Risk Factor Assessment

The null logistic model was extended to explore potential risk factors for *N. caninum* seropositivity. Risk factors were identified from questionnaire and open-source environmental and demographic data. These included: village-level livestock production system; household- and village-level dog ownership; feeding parturient materials from cattle to dogs; wildlife contact; environmental conditions expected to influence *N. caninum* oocyst survival; household management of grazing; herd size; household ownership of small ruminants and chickens; cattle introductions in the past 12 months; and local cattle population density. Village-level livestock production system was defined as “pastoral” (the majority of livelihoods based primarily on livestock production) and “mixed” (the majority of livelihoods based on a mixture of livestock and crop production) by local experts (district veterinary officers). The village-level dog ownership variable was the median number of dogs kept by households surveyed in each village. A number of potential proxies for wildlife contact were used. These were: farmer reports of observing any wild ungulate or carnivore (since wild canid observations were very rare) over the past 12 months; whether the household was within a wildlife area (conservation area, game controlled area, game reserve, national park, nature reserve, or wildlife management area) according to the world database on protected areas (UNEP-World Conservation Monitoring Centre, https://protectedplanet.net/); and the proportion of an 80 km area surrounding households (a circle with 5 km radius) that was classified as shrub or forest land (NASA Landsat Program, 2003, http://glcf.umd.edu/data/landsat/). Environmental variables that were hypothesized to influence oocyst survival were annual mean temperature and the average precipitation in the wettest quarter of the year (41), the clay, sand and organic carbon content of soil (42). Altitude was derived from shuttle radar topography mission data (43). Grazing management was split into two categories: restricted grazing in which cattle were tethered on pasture around the household or zero grazing in which fodder is brought to confined animals, and extensive grazing with a herdsperson. Local cattle population density was extracted at the household-level from the Food and Agriculture Organization's 2010 gridded livestock of the world data (44). All spatial data were manipulated in QGIS (version 2.14.3). Individual-level risk factors were cattle age (<18 months or ≥ 18 months on the basis of dentition), sex, and breed (indigenous or improved dairy cross).

The relationship between potential risk factors and individual level *N. caninum* seropositivity was first examined using univariable logistic regression. Given the large number of potential predictors and the fact that several of these predictors were included to represent similar features (e.g., wildlife contact, soil characteristics, etc.), we performed model selection. We used a Bayesian model averaging (BMA) approach for model selection (45). Model averaging was performed using an indicator variable with the Gibbs variable selection formulation (46). Briefly, this involves including a latent indicator variable (*w*) for each variable (*m*) in the model, *w*_*m*_. In a Bayesian context, the value of *w*_*m*_ is 1 if the linear predictor includes *m* and 0 if it does not. Hence, the posterior estimate for *w*_*m*_ represents the probability of inclusion of a particular variable in the regression equation and therefore an indication of its importance in explaining observed variation in the outcome of interest (i.e., the proportion of times the variable contributes to the posterior estimate). The model averaged co-efficients for predictors represent a sample from all possible models that are defined by all possible combinations of *w* indicator variables (47). Where *w*_*m*_ is close to 0, the co-efficient for *m* will also be shrunken toward 0, where *w*_*m*_ is close to 1, its effect will be preserved.

Given the low expected sensitivity of the diagnostic assay used, the null, univariable and multivariable logistic regression models were adjusted for test performance using the following formula (35):

$$\begin{array}{l} pa_{i}=p_{i}\;\times \;Se+\left(1-p_{i}\right)\;\times \;\left(1-Sp\right) \end{array}$$

Where *pa*_*i*_ is probability of a positive test result in individual *i* (i.e. the “true” seroprevalence) given the predicted probability of being seropositive, *p*_*i*_ (the “observed” seroprevalence), and the sensitivity (*Se*) and specificity (*Sp*) of the diagnostic test. We used positives and negatives defined using the conservative S/P cut-off, and therefore an estimated *Se* of 0.587 and *Sp* of 0.999 for adjustment.

Null, univariable and multivariable logistic regression models were run in JAGS via the *R2jags* package (48). Random effects were included at the household and village-level in all models. Weakly informative normal priors were used for all fixed and random effects. Convergence after a minimum burn-in of 50,000 and at least 100,000 iterations with a thinning interval of 20 was assessed by visual examination of three MCMC chains. The log of the number of cattle owned by a household was used on the expectation of a non-linear relationship with *N. caninum* seropositivity. All continuous predictors were standardized to have a mean of zero and standard deviation of one. Pairs of continuous variables were examined for collinearity using a Spearman's rank correlation coefficient: one of a correlated pair (ρ > 0.65) was excluded based on relative biological importance. Assessment of goodness of fit for the final multivariable model selected by BMA was made using a posterior predictive check (the “Bayesian *p*-value”) (49). This involves a comparison of the sum of the observed squared Pearson's residuals with the sum of squared Pearson's residuals expected from a distribution matching that specified by the model under assessment. Values close to 0.5 (and away from 0 and 1) suggest reasonable model fit (47). The Moran's I statistic for the null logistic regression model was calculated from village-level residuals using the *ape* package (50) in R.

#### Assessment of Disease Impacts

A mixed effects negative binomial regression was used to examine the relationship between the reported number of cattle abortions over the past 12 months and the within-herd prevalence of exposure to *N. caninum*. The log of the number of female cattle owned by the household at the time of the survey was included as an offset so that the abortion rate was modeled. Village was included as a random effect. Village-level production system was included to control for potential confounding. Negative binomial models were run in JAGS using the settings described above. Adjustment for test misclassification was not performed, instead we compare results derived using the conservative (24) and liberal (18.3) S/P cut-offs. Goodness of fit for models using each cut-off was assessed using the Bayesian *p*-value, as described above.

## Results

**Figure 1:**
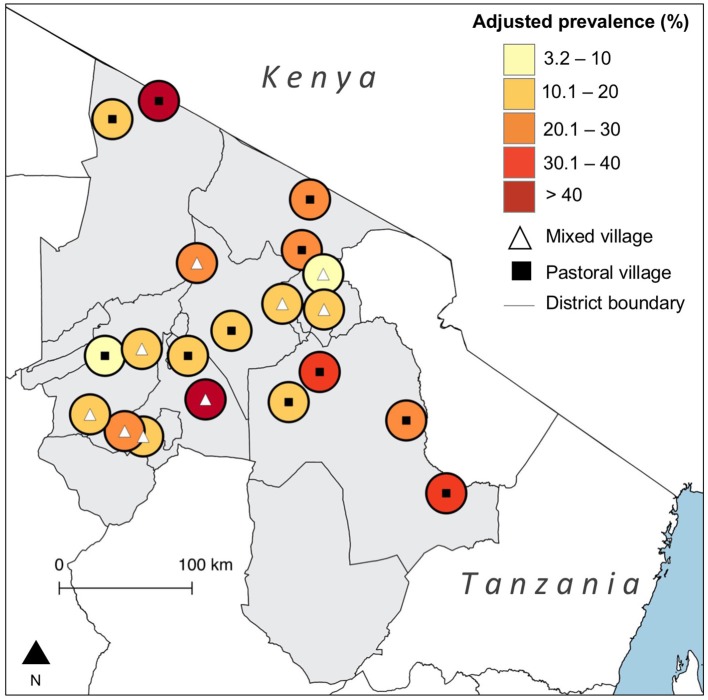
Map showing the village-level prevalence of *Neospora caninum* seropositivity in northern Tanzania with adjustment for diagnostic test performance. (Created using QGIS version 2.14.3, shapefiles from https://gadm.org/).

We tested 3,015 cattle serum samples, out of which 379 [12.6%, 95% Confidence Interval (CI) 11.4–13.8] were seropositive for *N. caninum* antibodies. Adjustment for test performance resulted in a true seroprevalence of 21.5% (95% CrI 17.9–25.4). On the basis of the liberal cut-off, the observed prevalence was 22.0% (95% CI 20.5–23.5), and the true prevalence was 25.3% (95% CrI 21.1–29.7). Of the 380 households sampled, 186 (49.0%, 95% CI 43.8–54.1) had at least one seropositive animal. This was 67.9% (95% CI 62.9–72.5) on the basis of the liberal cut-off. There was substantial variation in the true prevalence of infection between villages [3.2% (95% CI 0.3–9.2) to 60.3% (95% CI 43.6–79.1)] (Figure 1). Observed and true village-level prevalence estimates are provided in the Supplementary Materials.

The MOR and the ICC at the household level were 4.2 (95% CrI 3.0–6.7) and 39.9% (95% CrI 28.3–54.8), respectively; the MOR and ICC at the village level was 2.8 (95% CrI 1.9–4.8) and 19.7% (95% CrI 8.9–38.2), respectively. To put the household MOR into context, we would expect that, all else being equal, when comparing cattle in two different households anywhere in the study area, the odds of *N. caninum* seropositivity would be, in median, over four times higher for an animal in the household with higher within-herd prevalence than for an animal in the household with lower within-herd prevalence. In terms of ICC, we can say that around 40% of the differences in individual animal *N. caninum* exposure risk are at the household-level. Both of these measures suggest high levels of clustering of infection risk at the household level. Village was less important in structuring variation in infection risk. There was no evidence in autocorrelation (and therefore spatial clustering) in village-level residual odds of seropositivity (Moran's I = −0.08, *p* = 0.57).

The number and proportion of *N. caninum* seropositive samples and associated univariable odds ratios (OR) in relation to each categorical variable is shown in Table 1. A description of the continuous variables and the association with *N. caninum* seropositivity is shown in Table 2. We were able to conduct questionnaires in 360 households, representing 2,838 individual animals. Annual mean temperature was very strongly inversely correlated with altitude (ρ = −0.99). Altitude can be expected to be linked to a range of environmental effects, including temperature, and we therefore use altitude as the predictor of interest. Sand content of soil was inversely correlated with silt (ρ = −0.80) and clay (ρ = −0.95) content. While all soil properties can be expected to influence moisture content, which in turn can be expected to influence oocyst survival, we use sand content of soil in our multivariable analysis to reflect relatively high levels of water filtration and relatively low levels of water saturation (i.e., drier soils).

**Table 1:** Individual and household-level characteristics of categorical variables and their relationship with the seroprevalence of *Neospora caninum* in cattle in northern Tanzania.

**Risk factor**		**Total *N* (%)**	***Neospora* seropositive *n* (%)**	**Univariable regression**
				**OR (95% CI)**
**Individual-level**				
Age	<18 months	835 (29.4)	70 (8.4)	Ref
	≥18 months	2,003 (70.6)	285 (14.2)	2.49 (1.73–3.69)
Breed	Indigenous	2,567 (90.5)	334 (13.0)	Ref
	Cross	271 (9.5)	21 (7.7)	0.61 (0.24–1.55)
Sex	Female	1,894 (66.7)	248 (13.1)	Ref
	Male	944 (33.3)	107 (11.3)	0.79 (0.56–1.10)
**Household-level**				
Keep chickens	No	412 (14.6)	41 (10.0)	Ref
	Yes	2,404 (85.4)	312 (13.0)	1.15 (0.63–2.15)
Keep small ruminants	No	207 (7.3)	28 (13.5)	Ref
	Yes	2,631 (92.7)	327 (12.4)	0.85 (0.40–1.81)
Keep dogs	No	801 (28.2)	98 (12.2)	Ref
	Yes	2,037 (71.8)	257 (12.6)	1.03 (0.62–1.70)
Feed placenta to dogs	No	463 (16.3)	66 (14.3)	Ref
	Yes	2,375 (83.7)	289 (12.2)	0.88 (0.49–1.59)
Cattle introduction	No	1,974 (69.6)	246 (12.5)	Ref
	Yes	864 (30.4)	109 (12.6)	1.09 (0.70–1.71)
Restricted grazing	No	2,637 (93.0)	349 (13.2)	Ref
	Yes	198 (7.0)	6 (3.0)	0.22 (0.07–0.65)
Production system	Mixed	1,223 (43.1)	148 (12.1)	Ref
	Pastoral	1,615 (56.9)	207(12.8)	1.15 (0.40–3.41)
Wildlife area	No	1,477 (52.5)	163 (11.0)	Ref
	Yes	1,339 (47.5)	190 (14.2)	1.50 (0.67–3.49)
See wildlife	No	1,091 (38.4)	130 (11.9)	Ref
	Yes	1,747 (61.6)	225 (12.9)	1.36 (0.79–2.36)

**Table 2:** Household-level characteristics of continuous variables and their relationship with the seroprevalence of *Neospora caninum* in northern Tanzania.

**Risk factor**	**Median, mean (range)**	**Univariable regression**
		**OR (95% CrI)**
Number of dogs in village	2.00, 1.65 (0.00–3.00)	1.11 (0.78–1.58)
Cattle number	20, 63.96 (1.00–1,200)	1.15 (0.66–1.98)
Local cattle population density	0.8, 40.30 (0.2–5,820)	0.55 (0.34–0.87)
Sand content of soil (%)	48, 49 (31–66)	0.95 (0.60–1.44)
Organic content of soil (%)	14, 15 (1–55)	1.42 (1.07–1.89)
Clay content of soil (%)	33, 32.63 (18.00–47.00)	0.99 (0.65–1.57)
Silt content of soil (%)	18, 18.89 (11.00–28.00)	1.03 (0.71–1.51)
Precipitation of wettest quarter (mm)	406, 420 (251–719)	1,02 (0.64–1.71)
Mean annual temperature (°C)	19.5, 19.3 (14.5–24.1)	0.97 (0.58–1.62)
Altitude (m)	1,410, 1,470 (610–2,420)	1.06 (0.62–1.82)
Shrub or forest land (%)	0.12, 0.19 (0.00–0.99)	1.59 (1.08–2.38)

### Risk Factors for *N. caninum* Seropositivity

The outputs from the BMA procedure are summarized in Table 3. Variables with a probability of inclusion >0.5 were age >18 months; the local cattle population density; the percentage cover of shrub or forest land in the environment surrounding a household; and household use of restricted grazing. Older animals had more than two times the odds of being *N. caninum* seropositive compared to younger animals [Odds ratio (OR) = 2.17, 95% CrI 1.45–3.26]. While there was no evidence for a relationship with household herd size, local cattle population density was negatively associated with *N. caninum* seropositivity (OR = 0.69, 95% CrI 0.41–1.00). Cattle in households that reported using restricted grazing had reduced odds of seropositivity (OR = 0.72, 95% CrI 0.25–1.16). The credibility intervals for this variable broadly overlap one, so while it can be considered a moderately important predictor in explaining variation in *N. caninum* seropositivity (with a 0.63 probability of being in the model), the evidence for the size and direction of the effect should be considered weak. There was no evidence of a relationship between farmer reports of observing wildlife in the past 12 months or household location within a wildlife area and *N. caninum* positivity, but cattle kept in households in areas with a high percentage of shrub or forest cover were more likely to be *N*. *caninum* seropositive (OR 1.37, 95% CrI 1.00–2.14). There was no evidence for a difference in *N. caninum* seropositivity between production systems.

**Table 3:** Risk factors to *Neospora caninum* in cattle in northern Tanzania selected using Bayesian model averaging.

	**Indicator variable**	**OR**	**95% CrI**
Age	1.00	2.17	1.45–3.26
Local cattle population density	0.81	0.69	0.41–1.00
Shrub or forest land	0.78	1.37	1.00–2.14
Restricted grazing	0.62	0.72	0.25–1.16
Production system (pastoral)	0.48	0.89	0.36–1.51
Breed (cross)	0.39	0.95	0.51–1.50
Feed placenta to dogs	0.39	0.95	0.60–1.28
Keep small ruminants	0.38	0.99	0.64–1.52
Wildlife area	0.36	1.03	0.67–1.78
Keep chickens	0.36	1.05	0.79–1.67
Number of dogs in village	0.36	1.06	0.84–1.66
See wildlife	0.33	1.02	0.74–1.49
Keep dogs	0.31	1.00	0.73–1.36
Sex (male)	0.28	0.98	0.75–1.17
Cattle introduction	0.27	1.01	0.78–1.33
Organic carbon	0.19	1.01	1.00–1.05
Sand content of soil	0.05	1.00	1.00–1.00
Altitude	0.02	1.00	1.00–1.00
Precipitation of wettest quarter	0.01	1.00	1.00–1.00
Cattle number	0.00	1.00	1.00–1.00

### Assessment of Disease Impacts

The reported number of abortions in the past 12 months in study households ranged from 0 to 162. The seroprevalence of *N. caninum* in herds with at least one positive animal ranged from 8 to 100%, with an average of 24.6% (28.6% using the more liberal cut-off). There were five households in which the number of reported abortions over the past 12 months exceeded the number of adult female animals present at the time of the survey. We treated these as having 100% abortion rates (i.e., reduced the number of abortions to match the number of females). The multivariable negative binomial regression resulted in an incidence rate ratio (IRR) of 1.03 (95% CrI 1.00–1.06). Hence, for every 10% increase in within-herd *N. caninum* seroprevalence, the rate of abortion could be expected to increase by around 1.3 times. Production system was strongly associated with abortion rate, with this being considerably higher in households in pastoral villages than in mixed villages (IRR = 16.7, 95% CrI 3.6–133.5). The positive relationship between abortion rate and within-herd *N. caninum* prevalence was observed when the five households with 100% abortion rates were excluded from the dataset (IRR = 1.02, 95% CrI 1.00–1.05). There was not an important difference in results derived using the conservative and liberal cut-offs (data not shown). The Bayesian p-value for negative binomial models using different ELISA cut-offs ranged between 0.4 and 0.47.

## Discussion

In this study, we report an overall prevalence of *N. caninum* seropositivity of 21.5% among cattle in northern Tanzania. While the seroprevalence of *N. caninum* exposure varies between study villages and appears to be linked to environmental and demographic conditions, we find no evidence for a difference in prevalence between pastoral and mixed production systems. The moderately high seroprevalence we observe suggests neosporosis is likely to be an important cause of reproductive losses in cattle in northern Tanzania. Indeed, we find evidence for a positive association between within-herd *N. caninum* seroprevalence and abortion rates. A recently published study in neighboring Kenya provides further support for the importance of *N. caninum* as a cause of abortion in the region, with seropositivity of the pathogen reported to be associated with a greater proportion of fetal loss than either *Brucella* spp. or bovine viral diarrhea virus (32).

This is not the first study to report evidence for *N. caninum* infection in Tanzania. Barber et al. reported a seroprevalence of 22% in dogs in 1997 (51). A previous study in cattle in northern and north-eastern areas of Tanzania reported a seroprevalence of 8.1% in 2003 (33). However, the sample size was low and limited in its geographic coverage and it is therefore unclear whether the higher true prevalence reported in this study represents an increase in seroprevalence in northern Tanzania. A larger study conducted in the southern highlands (around 700 kilometers from our study area) in 2017 reported a seroprevalence of 4.5% (34). We found no evidence for an association between individual cattle *N. caninum* seropositivity and household- or village-level dog ownership, or with households reporting feeding placenta to dogs. The absence of a relationship with dog ownership was also reported from the southern highlands of Tanzania (34). Infection can be maintained in cattle populations by transplacental transmission (52), but there is no reason to suspect that dogs do not act as reservoirs of *N. caninum* for cattle in Tanzania, and a high seroprevalence of infection has been found in dogs in both Kenya and Tanzania (25, 32, 33, 51). The lack of an observable effect for household-level dog ownership may point to the importance of contamination of grazing areas by free roaming dogs. Dogs in Tanzania are owned by specific households, but often roam far during the day (53). While we did not find a relationship with village-level dog ownership, it could be expected that free-ranging dogs infected with *N. caninum* could contaminate grazing areas across a wide area, thereby potentially exposing cattle from multiple households to oocysts shed by a single dog. This mechanism is thought to be important for the transmission of other dog-mediated pathogens to livestock in northern Tanzania (54).

Our data provide evidence for a negative relationship between cattle population density and *N. caninum* seropositivity in northern Tanzania. The biological explanation for this relationship is unclear, particularly since cattle population density is strongly correlated with human population density (55) which, in turn, tends to be correlated with dog population density (56). Dog population density has been found to predict *N. caninum* seropositivity in other settings (57). The observed negative effect with cattle population density in this study may represent a lack of confounding control by production system. In our study area, small holder production systems (i.e., mixed crop and livestock, with small cattle herd sizes) are found primarily in peri-urban areas with high human and cattle population density. These are also the areas in which restricted grazing predominates (none of the pastoral households in our study reported restricted grazing). It could therefore be expected that cattle reared in small holder households are at lower risk of *N. caninum* exposure than cattle reared in pastoral households, which are found in low cattle population density areas and practice extensive grazing. The lack of an observable effect by production system in our study (and the potential lack of control for the effect of cattle population density) may be due to the non-specific nature of the definitions used. Our mixed farming category includes both small-holder and agro-pastoral households. Agro-pastoral households practice mixed crop and livestock production but tend to have larger herd sizes and are found in more rural, low cattle population density locations than small holder households in our study area. Livestock reared in agro-pastoral households could therefore be expected to have a different *N. caninum* risk than those reared in small holder households. Further work to explore the effect of production system on *N. caninum* risk in Tanzania, including better control for the range of livestock production systems that exist in the region, would be valuable.

While we did not find evidence for a relationship between either cattle being reared in a wildlife area or farmer reports of seeing wildlife in the past 12 months and *N. caninum* seropositivity, we did find evidence for a strong association with levels of forest and shrub cover in the area surrounding households. It could be hypothesized that such areas would support the largest wildlife populations, and particularly small and medium sized members of the *Carnivora* order. We are not aware of any studies that have directly evaluated the role of wildlife as reservoirs for *N. caninum* in cattle in Tanzania, but serological studies have demonstrated positivity in cheetah and spotted hyena in Kenya (26). These wild carnivores, among others, are found in northern Tanzania, particularly in pastoral settings. Sylvatic cycles have been demonstrated in other settings, including in the Australian dingo (1, 2), water buffalo (58) as well as those involving rodents (59–61). Further work to explore the role of wildlife in the epidemiology of *N. caninum* in Tanzania is recommended. We included the forest and shrub cover variable to represent wildlife habitat suitability, however alternative explanations for its effect on *N. caninum* exposure risk should also be considered. These include the reduced availability of grassland in forest and shrub areas, resulting in greater concentration of cattle grazing in smaller areas. Alternatively, while we did not find a relationship with precipitation, altitude or soil type, the microclimatic conditions that are particular to forest and shrub areas may favor *N. caninum* oocyst maturation and survival. Unsporulated *N. caninum* oocysts are said to be highly resistant in the environment (62) and are thought to survive for several years (3). However, limited work has been conducted on the impact of macro or micro-climatic conditions on oocyst survival or rates of maturation (3), particularly in the African context.

We observed that animals >18 months were more likely be *N. caninum* seropositive than juvenile animals. A similar relationship with age has been reported widely (32, 63, 64). Cattle are infected with *N. caninum* for life, and this effect is likely to represent the cumulative exposure risk to sporulated oocysts in the environment as animals age (3).

There are several limitations to our study that should be considered. While we find weak evidence for a relationship between within-herd seroprevalence and abortion rate within a household, it should be noted that these seroprevalence estimates are based on a maximum sample of 10 animals per household. Estimates of within-household seroprevalence are therefore based on small sample sizes and associated with very low precision. Additionally, these data are likely to be strongly influenced by recall bias. This is likely to be a particular issue for abortions associated with *N. caninum*, which tend to occur in the second trimester and may therefore be missed or not recollected by livestock keepers. Cattle breeding in the study area is often unplanned and pregnancy diagnosis rare, hence it is likely that only a proportion of abortions will be noticed and reported by participating farmers. Data collection followed a central point procedure in which farmers were invited to attend the sampling event and which may therefore have introduced selection bias: any farmer who did not attend was not included in the sample. We sought to reduce this as much as possible by running several sampling events at different points within the same village and by linking sampling with village-level disease control to incentivize attendance. Finally, while the focus of this study was on cattle, there is growing evidence that sheep and goats can be affected by neosporosis (5, 9–12). Since the majority of cattle-keeping households in rural Tanzania also keep small ruminants, and the majority of these are freely grazed on communal grazing lands, there is a great need for future studies in the country to incorporate sheep and goats into assessments of the epidemiology and impacts of *N. caninum*.

Our study results have a number of implications for disease control in Tanzania. Despite the moderately high prevalence of infection detected in this study, we are not aware of the availability of routine testing for neosporosis in either government or commercial laboratories in Tanzania. Provision of such testing would assist farmers and veterinarians with herd health planning and may be particularly valuable for the growing dairy industry in Tanzania. We find some evidence of a relationship between within-herd seroprevalence and herd-level abortion rates, suggesting that the control of *N. caninum* could contribute to reduced reproductive losses among cattle in the region. Recent research from northern Tanzania demonstrates that cattle abortions are negatively associated with schooling expenditure and positively associated with food expenditure (65). *Neospora caninum* infection and associated abortions can be expected to contribute to this negative impact on household welfare. Our results indicate several potential control points. We find some evidence that restricted grazing is associated with reduced risk of *N. caninum* infection and may therefore be a strategy cattle-keepers can use to reduce their abortion risk. It is important to note, however, that restricted grazing requires greater resources in terms of labor and the purchase or collection of fodder. This management system is therefore likely to be impractical for many households, and particularly those in pastoral systems, with large herds relying on extensive grazing in often marginal environments. Reducing contamination of the environment with dog feces could also be expected to contribute to reduced infection risk. Such an approach may be possible in areas where dogs are well-controlled, but in the presence of free ranging dogs, preventing contamination of communal grazing areas is likely to be challenging. Village dogs in these settings may also play an important role in reducing contamination of the grazing environment by deterring the wild canids from the grazing areas close to community settlements. Overall, further work is required in Tanzania, and East Africa more broadly, to explore approaches that can be used, and their applicability to different production systems, in order to control this economically important parasite.

## Conclusion

*N. caninum* seropositivity is moderately common in cattle in northern Tanzania and is likely to be a cause of abortion. We find some evidence that management practices, such as restricted grazing, reduce the risk of infection, suggesting contamination of communal grazing areas may be an important source of infection. Evidence of relationships between livestock seropositivity and shrub and forest habitats may also suggest a role for wildlife in the epidemiology of *N. caninum* in Tanzania that would be a valuable area for future study. To date, limited research has been conducted on the epidemiology and control of *N. caninum* in East Africa, but this parasite is likely to be an important cause of abortions and thus an economically important parasite to monitor and control.

## Data Availability Statement

The datasets generated for this study are available on request to the corresponding author.

## Author Contributions

GS and WG performed data analysis and manuscript drafting. TK, ES, and WG performed data collection. CH, KT, KA, EI, and FK performed laboratory analysis. SC acquired funding. KK, FL, JB, JC, WG, and SC provided supervision. All authors reviewed and provided input into the manuscript.

### Conflict of Interest

The authors declare that the research was conducted in the absence of any commercial or financial relationships that could be construed as a potential conflict of interest.
