# Supplementary material for: The Sero-epidemiology of Neospora caninum in Cattle in Northern Tanzania
Source: Front Vet Sci. 2019 Sep 26;6:327. doi: 10.3389/fvets.2019.00327 (PMC6798052; doi:10.3389/fvets.2019.00327)
Supplement: Supplementary file 1 [file Table_1.DOCX]

***Supplementary materials***

Table S1 shows values for the village level seroprevalence of antibodies to *Neospora caninum* in the study area.

| **Village** | **District** | **Region** | **Production system^1^** | **Number sampled** | **Observed prevalence^2^** | **95% confidence interval** | **True prevalence^3^** | **95% credibility interval** |
| --- | --- | --- | --- | --- | --- | --- | --- | --- |
| Arri | Babati | Manyara | M | 64 | 5.9 | 1.9 - 15.1 | 13.0 | 4.2 - 26.1 |
| Endanyawish | Mbulu | Manyara | P | 102 | 1.0 | 0.1 - 6.1 | 3.2 | 0.3 - 9.1 |
| Engikaret | Longido | Arusha | P | 197 | 13.2 | 9.2 -18.5 | 26.9 | 18.1 - 37.6 |
| Engusero sambu | Ngorongoro | Arusha | P | 152 | 24.8 | 19.1 - 31.4 | 57.3 | 42.8 - 74.2 |
| Ilkerin | Arusha | Arusha | M | 93 | 7.9 | 3.7 -15.5 | 16.4 | 7.3 - 28.5 |
| Kansay | Karatu | Arusha | M | 225 | 9.3 | 6.1 -13.8 | 18.2 | 11.5 - 26.5 |
| Kimokowa | Longido | Arusha | P | 176 | 10.7 | 6.9 -16.0 | 21.2 | 13.1 - 31.1 |
| Kisimiri | Meru | Arusha | M | 101 | 1.9 | 0.3 -7.5 | 4.9 | 0.9 - 11.9 |
| Komolo | Simanjiro | Manyara | P | 126 | 15.4 | 10.2 - 22.5 | 32.2 | 20.9 - 46 |
| Long | Babati | Manyara | M | 175 | 12.1 | 8.0 - 17.6 | 24.4 | 15.7 - 35.2 |
| Lositete | Karatu | Arusha | M | 135 | 12.3 | 7.8 -18.8 | 25.1 | 15.3 - 37.4 |
| Maheri | Mbulu | Manyara | M | 241 | 9.4 | 6.3 -13.7 | 18.4 | 11.8 - 26.4 |
| Minjingu | Babati | Manyara | P | 144 | 5.3 | 2.5 - 10.5 | 10.5 | 4.7 - 18.7 |
| Naiti | Monduli | Arusha | P | 197 | 9.6 | 6.2 - 14.5 | 19.1 | 11.7 - 28.1 |
| Nambala | Meru | Arusha | M | 136 | 6.2 | 3.1 - 11.8 | 12.4 | 5.8 - 21.2 |
| Ngage | Simanjiro | Manyara | P | 170 | 11.9 | 7.9 - 17.5 | 24.1 | 15.3 - 35.1 |
| Oloipiri | Ngorongoro | Arusha | P | 144 | 7.7 | 4.2 - 13.3 | 15.2 | 8.1 - 24.7 |
| Ruvu remitii | Simanjiro | Manyara | P | 164 | 14.6 | 10.1 - 20.6 | 30.2 | 20.2 - 42.3 |
| Sarame | Babati | Manyara | M | 113 | 25.7 | 19.1 - 33.5 | 60.3 | 43.6 - 79.1 |
| Sukuro | Simanjiro | Manyara | P | 160 | 6.4 | 3.5 - 11.5 | 12.7 | 6.6 - 20.8 |

**Table S1. Village level observed and true prevalence of antibodies to *Neospora caninum* in northern Tanzania**

^1^ M = Mixed; P = pastoral; ^2^ Estimated using an S/P cut-off of 25; ^3^ With adjustment for diagnostic test performance
